# Triple trouble: Understanding the burden of child undernutrition, micronutrient deficiencies, and overweight in East Asia and the Pacific

**DOI:** 10.1111/mcn.12950

**Published:** 2020-08-24

**Authors:** Jessica L. Blankenship, Christiane Rudert, Victor M. Aguayo

**Affiliations:** ^1^ Nutrition Section UNICEF East Asia and the Pacific ReFgional Office Bangkok Thailand; ^2^ Nutrition Section, Programme Division UNICEF New York New York

**Keywords:** child dietary quality, child dietary quality, maternal nutrition, micronutrient, overweight, stunting, wasting

## Abstract

Young children in the East Asia and the Pacific region^1^ are failing to thrive, in large numbers, as indicated by stagnation in the decline of stunting, wasting, and micronutrient deficiencies and the fastest growing rates of overweight in the world. Eliminating the triple burden of malnutrition is essential to ensure that, as a matter of right, all children reach their full physical growth and development potential and actively contribute to equitable prosperity and the sustainable development of their communities and nations. Ending all forms of malnutrition will only be achieved through the implementation of effective policies and programmes soundly based on an understanding of the leading contextual drivers of child malnutrition. To address the lack of data on these drivers in the region, the UNICEF regional office for East Asia and the Pacific commissioned a series of papers in 2017–2019 to fill gaps in the current body of evidence on the triple burden of maternal and child malnutrition. This series includes analyses of the determinants of child malnutrition including maternal nutrition status, dietary quality of children, inequity, and poverty. Additionally, policy and programmatic actions associated with improved coverage and quality of nutrition interventions are reviewed. This overview paper summarizes the findings of these analyses and presents recommendations for the direction of future advocacy, policy, and programmatic actions to address the triple burden of malnutrition in East Asia and the Pacific.

**Key messages**

- East Asia and Pacific region has a well established triple burden of malnutrition with stagnation in the reduction of stunting,wasting and micronutrient deficiencies and the fastest growing rates of overweight in the world.
- Indicators of poverty and inequity are leading drivers to child malnutrition with children often exposed to multi‐dimensional forms of poverty.
- Poor maternal nutrition is a consistent predictor of stunting and wasting across the region.
- Multisectoral integrated interventions across the health, food, social protection, WASH and education sectors are needed to eliminate undernutrition in children while preventing rises in childhood overweight in East Asia and the Pacific.

## 1 INTRODUCTION

In East Asia and the Pacific, robust economic growth and improved access to essential health services has resulted in dramatically lower mortality rates for young children (You et al., 2015). Despite more children surviving, millions of young children in the region are failing to thrive as indicated by stagnation in reduction of stunting, wasting, and micronutrient deficiencies and the fastest growing rates of overweight and obesity in the world. The latest global estimates indicate that the region is home to 13 million under 5‐year‐olds who are stunted, 4.5 million who are wasted, and 9.7 million who are overweight (i.e., 9% of global burden of stunting and wasting and 24% of the global burden of overweight; UNICEF, 2019). Additionally, an estimated 46% of children in Southeast Asia and 33% of children in the Pacific suffer from micronutrient deficiencies (UNICEF, 2019). The triple burden of malnutrition—undernutrition, micronutrient deficiencies, and overweight—has multiple drivers; these include inadequate maternal nutrition, consumption of nutrient‐poor diets in infancy and early childhood, and changing food systems that bring about increasing exposure to cheap and convenient sugary beverages and unhealthy foods high in salt, sugar, and fat but poor in essential nutrients (Popkin, Corvalan, & Grummer‐Strawn, 2019). Underlying factors include poor sanitation, water quality, poor hygiene practices, combined with sociocultural factors, inequity, and poverty. A major new factor that is likely to contribute to rising rates of malnutrition is the COVID‐19 pandemic, which is exacting a major economic toll on vulnerable households.

Children who are undernourished early in life experience worse health, growth, and developmental deficits during childhood as well as lower productivity and poorer livelihoods in adulthood, leading to a loss of human capital (Black R., et al., 2013). In addition, children who are stunted or wasted during the first 1,000 days are likely to undergo metabolic and physiological changes in response to undernourishment, increasing their susceptibility to becoming overweight and developing diet‐related noncommunicable diseases later in childhood and into adolescent and adulthood (Wells et al., 2019).

Development of targeted actions to address the triple burden of malnutrition in children needs to be based on a sound understanding of the leading drivers of child malnutrition and the policies and programmes needed. To address the lack of data on drivers of the triple burden of child and maternal malnutrition, the UNICEF regional office for East Asia and the Pacific commissioned a series of papers in 2017–2019. This series includes analyses of the underlying and basic causes of child malnutrition, including dietary quality of children, maternal nutrition status, inequity, and poverty. Additionally, policy and programmatic actions associated with improved coverage and quality of nutrition interventions are reviewed. The 12 articles included in this special issue of *Maternal and Child Nutrition* summarize the findings of these analyses.

## 2 THE EAST ASIA AND THE PACIFIC REGION IS ENCUMBERED BY THE TRIPLE BURDEN OF MALNUTRITION

The triple burden of child malnutrition takes many forms in East Asia and the Pacific and often coexists in the same population, household, and even in the same child. Using thresholds by the Global Nutrition Report 2018 on the triple burden of malnutrition, the majority of the 26 countries in the region have a double burden of stunting, overweight, or anaemia (16), and 6 countries suffer a triple burden (Development Initiatives, 2018). Further, these high numbers are likely underestimated due to insufficient data in the Pacific.

Discordance in nutritional status as measured by the maternal child double burden (MCDB) is increasing in East Asia and the Pacific and is found in all countries with data (Popkin et al., 2019). Rachmi, Li, and Baur (2018) found MCDB ranging between 5% in Vietnam and 30% in Indonesia. Despite having the highest prevalence of adult overweight in the world, little is known about MCDB in the Pacific Island countries. In this issue, Blankenship, Cashin, Nguyen, and Ip (2020) found that in the Marshall Islands, a staggering 25% of households with a child under 5 years of age included a stunted child and an overweight or obese mother.

Few countries in East Asia and the Pacific have recent micronutrient surveys; however, those available indicate that micronutrient deficiencies are common among young children. In a review of micronutrient deficiencies in South East Asia, Wieringa, Dijkhuizen, and Berger (2019) found that over 30% of children under 5 years of age were deficient in zinc, whereas thiamine deficiency in children was endemic in Cambodia, Laos, Thailand, and Myanmar. In this issue, Janmohamed et al. (2020) present the first examination in the region of the prevalence of concurrent overweight and micronutrient status among children under 5 years in Mongolia. The authors found high prevalence of micronutrient deficiencies among overweight children with 11.8% deficient in vitamin A, 26.6% deficient in iron, and 55.1% deficient in vitamin D. The prevalence of micronutrient deficiencies was similar between overweight and nonoverweight children indicating discordance between high energy intake and micronutrient intake.

## 3 POVERTY AND INEQUITY

Globally, inequity, and deprivation are key drivers of child stunting (Argaw et al., 2019) with research from South Asia finding low levels of maternal education and household poverty to be key drivers of child stunting (Rama, Beteille, Li, Matra, & Newman, 2015). In East Asia and the Pacific, despite rapid economic growth, income inequity has increased in the last 25 years (Economic and Social Commission for Asia and the Pacific, 2018). Poverty is likely to be significantly exacerbated by the COVID‐19 pandemic particularly among vulnerable households. In this issue, nationally representative data was examined in Myanmar (Blankenship, Cashin, et al., 2020), the Marshall Islands (Blankenship, et al., 2020), and Thailand (Okubo, Janmohamed, Topothai, & Blankenship, 2020) to identify the key drivers of child malnutrition. Despite varying degrees of economic status and progression along the nutrition transition, in all three countries, the poorest households had the highest risk of child stunting, with the poorest households also having highest risk of wasting in Thailand. Other indicators of inequity, including lower maternal education (Thailand) and single motherhood (Marshall Islands), were associated with increased risk of stunting.

In their paper, Janmohamed et al. (2020) identified the association between poverty, dietary quality in early childhood, and child overweight in Mongolia. Child dietary quality was poor across all households, and prevalence of overweight was not associated with wealth. Even in severely food insecure households, 10% of children were overweight, and less than half of overweight children had an acceptable diet. In a trend that may apply more broadly to the region, children's inability to achieve an adequate diet in Mongolia was found to be primarily due to limited dietary diversity, rather than insufficient caloric intake.

Poor water, sanitation, and hygiene (WASH) practices are known drivers of malnutrition in children; chronic intestinal infections from poor WASH practices and contaminated water can lead to stunting, wasting, and anaemia through reduced appetite of children, reduced nutrient absorption, and increased nutrients losses (Dewey & Mayers, 2011). In this issue, the associations between WASH (improved sanitation facilities and improved drinking water) and undernutrition (child stunting and anaemia) were examined by Rah, Sukotjo, Badgaiyan, Cronin, and Torlesse (2020) in Indonesia. The authors found that access to improved sanitation facilities was associated with 29% reduced odds of being stunted, but no association was found with anaemia. Access to improved drinking water was not associated with reduced odds of stunting or anaemia.

One of the limitations in the measurement of improved drinking water is that the indicator takes into consideration the source of water but not water quality. Poirot et al. (2020) examined the contamination of drinking water with *Escherichia coli* and coliform in Cambodia both at the point of use and at the source of collection. The authors found that water quality worsened after storage in the household with 88% of improved water sources contaminated with coliform and 48% contaminated with *E. coli.* These findings call into question the assumptions about the quality of drinking water collected from widely acknowledged improved sources and may explain the poor association between improved water use and child malnutrition.

Poor dietary, health, and WASH practices are leading drivers of child malnutrition, and integrated multisectoral interventions are needed to address the multidimensional impacts of these factors on nutrition status. In this issue, Karpati, de Neubourge, Laillou, and Poirot et al. (2020) explored the effect of multidimensional poverty on child stunting in Cambodia with 78% of children under 5 years multidimensionally deprived and high overlap in deprivation among dietary intake, sanitation, water, and housing. In their paper, Laillou et al. (2020) examined the contribution of various predictors on child stunting and wasting prevalence in Cambodia. Poor child feeding practices, source of drinking water, and low household wealth were the strongest predictors of both stunting and wasting.

## 4 POOR MATERNAL NUTRITION

Poor maternal nutrition is a well‐known driver of child malnutrition. Women's nutritional status, both before and during pregnancy, impacts fetal growth and development; a child whose pregnant mother is short, thin, or anaemic is more likely to experience in utero growth restriction, preterm delivery, and low birth weight (Black R. , et al., 2013). Although indicators of maternal nutrition status, including mid–upper arm circumference (MUAC) under 23 cm, short maternal stature, and anaemia are known to be reliable indicators to identify the risk of low birth weight infants, the association between maternal nutrition status and child nutrition status has been largely unexplored in East Asia and the Pacific.

The globally consistent findings of a higher likelihood of stunting among children of shorter mothers suggests adverse intergenerational nutritional impacts on birth size and growth limiting the attainment of children's genetic height potential (de Onis & Branca, 2016). In this issue, research from Cambodia, the Marshall Islands, and Myanmar confirm global evidence that maternal short stature is a leading determinant of child stunting. Increased risk of child stunting with maternal height under 160 cm was found in both Myanmar (Blankenship, Cashin, et al., 2020) and the Marshall Islands (Blankenship et al., 2020), whereas Karpati, de Neubourg, Laillou, and Poirot (2020) confirmed a strong association between maternal height (<145 cm) and child stunting in Cambodia. Shorter maternal height was also the leading predictor of MCDB in the Marshall Islands. In both the Marshall Islands and Myanmar, shorter maternal height is highly prevalent with over a quarter (27.5%) of women in the Marshall Islands and 30.3% of women in Myanmar under 150‐cm tall.

The association between low MUAC during pregnancy and poor neonatal growth was explored in this issue by Kpewou et al. (2020) who found that infants had a 60% higher risk of suboptimal length for age at the age of 0 to 3.5 months if their mother had a MUAC < 23 cm during pregnancy. Although body mass index is not commonly measured throughout pregnancy in the region, in Myanmar, prevalence of maternal underweight after pregnancy was associated with 1.6 times higher risk of child wasting compared with mothers with normal nutrition status (Blankenship, Cashin, et al., 2020).

Few countries in Asia and the Pacific have reliable data on birth weight due to recall bias and high prevalence of missing data. In this issue, Okubo et al. (2020) found low birth weight to be the leading predictor of child stunting, concurrent stunting and overweight, and concurrent stunting and wasting in Thailand. The findings indicate that children born with low birth weight are at increased risk of not only undernutrition but also developing overweight. In Myanmar, Blankenship, Cashin, et al. (2020) found that the mother's perceived size of the child as smaller than normal at birth was associated with 2.1 times higher risk of stunting compared with children perceived by the mother to be of normal size.

## 5 NUTRITION POLICIES AND PROGRAMMES

Multisectoral‐integrated interventions are needed to prevent child malnutrition in East Asia and the Pacific. However, national policies and programmes for nutrition do not always reflect the need to address the key drivers of child malnutrition or the shift from a focus on undernutrition alone to addressing the triple burden of malnutrition. In this issue, Nguyen et al. (2020) found strong regional dedication in the Association of Southeast Asian Nations declaration to end all forms of malnutrition; however, the National Nutrition Strategies in place in countries do not adequately incorporate evidence‐based interventions, targets, and indicators. Although all countries included national policy objectives to improve the nutrition status of mother and children with a focus on undernutrition, only three of the nine countries included a policy objective to improve micronutrient status or to prevent and control overweight or diet‐related noncommunicable diseases. Additionally, although all nine countries included measurable indicators and targets for nutrition in their National Nutrition Strategies, key indicators from the Global Nutrition Targets 2025 were missing in several countries including low birth weight, overweight, wasting, and micronutrient deficiencies.

The presence of policies and strategies does not automatically translate into well‐implemented programmes. The complexity of implementing and maintaining effective programmes to address the triple burden of malnutrition is explored in this issue through two examples: universal salt iodization in Cambodia and breastfeeding protection, promotion, and support in China.

Salt iodization has been one of the great success stories for nutrition in East Asia and the Pacific. However, the region has experienced backsliding in iodizing salt and reducing iodine deficiency, Cambodia being one of the most critical examples. In their paper, Codling, Laillou, Rudert, Mam, and Gorstein (2020) review how sustainable funding and institutionalization of responsibilities by national governments and salt industry are vital for a successful salt iodization programme. The experience from Cambodia underlines the importance of legislation to make salt iodization mandatory, enforcement mechanisms to ensure programme implementation, and commitment by the private sector to ensure sustainability.

The necessity of multisectoral actions to address poor nutrition practices is explored by Li, Nguyen, Wang, Mathisen, and Fang (2020) in an examination of the individual, family, community, and health facility factors associated with inadequate breastfeeding practices in China. The authors found that poor breastfeeding practices were largely influenced by the environment, including a lack of support from partners and family members, poor delivery of breastfeeding counselling and support through the health system, and rampant marketing of breast milk substitutes. Factors outside the health sector were also key drivers of poor breastfeeding practices, including insufficient paid maternity leave, few breastfeeding friendly public spaces, and overall poor awareness and knowledge by women and their families about the benefits of breastfeeding.

## 6 ADDRESSING THE TRIPLE BURDEN IN A CHANGING FOOD ENVIRONMENT

The set of papers in this special issue brings together research on the drivers of the triple burden of malnutrition in East Asia and the Pacific, providing insights into the pathways to accelerate progress in the reduction of undernutrition and micronutrient deficiencies while halting the rise of overweight in both children and women. The findings from this special issue reveal five implications on the direction of future advocacy, policy, and programme actions.

### 6.1 First, the triple burden requires governments to address all forms of malnutrition in an integrated manner across the life cycle

A siloed approach has often been used to design strategies to address stunting, wasting, micronutrient deficiencies, and overweight in East Asia and the Pacific with stronger focus on the reduction of stunting over all other forms of malnutrition (Nguyen et al., 2020). However, these multiple forms of child malnutrition share common risk factors, including poor maternal nutrition and care practices, consumption of nutrient‐poor diets during the first 2 years of life, and socioeconomic deprivations including inadequate access to safe water and sanitation, poverty, and inequities. Silos must be broken down through a realignment of narratives, policies, and programmes across the life cycle to ensure that countries address maternal and child malnutrition in all its forms (Branca et al., 2019)

### 6.2 Second, improving women's nutrition is central to breaking the intergenerational triple burden of malnutrition in East Asia and the Pacific

Poor maternal nutrition is a key driver of child malnutrition; children born to short and undernourished mothers are more likely to be born with a low birth weight and significantly higher risk for stunting, wasting, and overweight later in life. Despite this, maternal nutrition interventions are most often delivered only through antenatal care programmes with variable coverage, quality, and intensity (de Silva, Untoro, Blankenship, & Udomkesmalee, 2019). Key bottlenecks to improving maternal nutrition include poor allocation of resources, lack of political commitment, and the scarcity of data on maternal nutrition, including on the coverage and quality of programmes aiming to improve it. A focus on the following strategies is needed: addressing all forms of maternal malnutrition through innovative methods for promoting social and behaviour change using novel channels of communication, strengthening antenatal care services with quality counselling and support, measuring appropriate pregnancy weight gain and managing insufficient and excessive weight gain, and preventing and treating multiple micronutrient deficiencies and anaemia.

### 6.3 Third, an integrated multisectoral approach is needed to address the multiple determinants to child malnutrition

No one system can address the multiple drivers of child malnutrition. A multisectoral approach is needed across food, health, water and sanitation, education, and social protection systems to address the key drivers of poor nutrition in children and women with synergistic impact (Hawkes, Ruel, Salm, Sinclair, & Branca, 2019). Policies, legislation, and fiscal measures related to the food system should increase the availability, access, and affordability of nutritious foods while providing disincentives for consumption of nutrient‐poor processed foods rich in sugar, salt, and fat, such as taxation, marketing restrictions, and clear front‐of‐pack labelling. The health system is a key platform for delivery of essential services and counselling to women and other caregivers on appropriate infant feeding and care practices and to reach women during pregnancy with timely nutrition counselling, support, and services. The water and sanitation system provides safe drinking water—a key component of a healthy diet—and safe sanitation services to improve the utilization of nutrients from the foods consumed. The education system is a platform for the delivery of nutrition services such as micronutrient supplementation, fortified foods, and deworming prophylaxis, for improvements in nutrition literacy and for the fostering of healthy food environments that promote good diets. Finally, social protection systems have the potential to alleviate the financial barriers to healthy diets and other essential social services and to support the adoption of positive nutrition practices. Integrated multisectoral approaches require effective government coordination among sectors, defined roles, responsibilities and accountabilities, and budgets.

### 6.4 Fourth, nutrition‐sensitive social protection programmes are needed to address the disparities and inequalities in child growth during the first 1,000 days

Social protection programmes, including nutrition‐sensitive cash transfers, are increasingly being used in East Asia and the Pacific to improve households' access to better diets, health services, and improved living environments. These programmes are being expanded and adjusted to cope with the impact of the COVID‐19 pandemic. Nutrition‐sensitive social protection has potential to reduce the inequity within populations and to address the higher prevalence of child stunting and other forms of malnutrition in the poorest households. However, these gains are not automatic and must be carefully integrated into the design and implementation of social protection programmes. To enhance the impact of social protection on maternal and child nutrition, nutrition objectives need to be part of the design of social protection programmes with a focus on the first 1,000 days and appropriate scope, frequency, duration, and timeliness to help households meet their needs for healthy child growth. Social protection programmes should also be used as an entry point for multisectoral interventions including increased access to and quality of nutrition, health, and WASH services; delivery of nutrition specific interventions such as improving affordability and access to healthy foods and diets, including fortified complementary foods, and the provision of multiple micronutrient powders for home fortification; and targeted access to livelihoods and women's empowerment interventions.

### 6.5 Fifth, prioritization is needed in all countries to collect, analyse, and utilize data to assess progress and to inform decisions

Countries in East Asia and the Pacific suffer from large data gaps for maternal and child nutrition indicators compounded by infrequent data collection in the Pacific Island countries. These gaps hinder the ability to understand context‐specific barriers, enablers, and pathways to address the triple burden of malnutrition. Improved use of routine information systems and timely surveys are needed to gather essential data on the following: anthropometric and micronutrient status of children and women; feeding practices in early childhood; dietary practices in middle childhood and adolescence, including exposure to unhealthy food environments and consumption of unhealthy foods; and access, coverage, and quality of essential nutrition services delivered by the health, water and sanitation, education, and social protection systems. The impact of the COVID‐19 pandemic on nutrition status, diets, services and practices will also need to be assessed. Analysis and interpretation of this data is needed to help prioritize policies and programmes, measure progress against targets, and advocate for increased funding and resources for cost‐effective interventions.

## 7 CONCLUSION

The East Asia and the Pacific region is encumbered by the triple burden of malnutrition, characterized by a stagnation in the reduction of child undernutrition and micronutrient deficiencies and a growing prevalence of overweight and obesity. This situation is exacerbated by growing socioeconomic inequities and by dynamically changing food systems that bring about both positive and negative impacts on children's diets. It is likely to be further exacerbated by the COVID‐19 pandemic. Children are at risk of multiple forms of malnutrition throughout the life cycle with common and interlinked drivers of stunting, wasting, micronutrient deficiencies, and overweight. Accelerating progress in reducing the burden of undernutrition and micronutrient deficiencies while halting the steady rise of overweight requires renewed focus on improving women's nutrition during adolescence, pregnancy, and breastfeeding; ensuring appropriate diets for infants and young children in the first 2 years of life; and addressing the underlying drivers of poverty, inequity, and lack of access to essential nutrition and other services, including safe drinking water and safe sanitation (Hawkes et al., 2019).

In order to address the drivers of the triple burden of maternal and child malnutrition, synergistic and accelerated change is needed through a broader and bolder multisectoral approach. The series of papers in this special issue of *Maternal and Child Nutrition* present evidence on key drivers in countries throughout the region; it is our expectation that they will support the efforts by all stakeholders to address the triple burden of malnutrition and to achieve sustainable development in all countries in East Asia and the Pacific.

## CONFLICTS OF INTEREST

J. L. B, C. R., and V. M. A. are members of the United Nations Children's Fund (UNICEF). The authors alone are responsible for the views expressed in this publication and declare that they have no conflicts of interest.

## CONTRIBUTIONS

The authors' responsibilities were as follows: Jessica L. Blankenship drafted this manuscript, and Jessica L. Blankenship, Christiane Rudert, and Victor M. Aguayo provided critical intellectual feedback to help revise the manuscript. All authors have read and approved the final manuscript.
